# Editorial for the Special Issue on the Application of Microfluidic Technology in Bioengineering

**DOI:** 10.3390/mi16091022

**Published:** 2025-09-05

**Authors:** Shuli Wang, Yigang Shen

**Affiliations:** 1 Fujian Engineering Research Center for Solid-State Lighting, Department of Electronic Science, School of Electronic Science and Engineering, Xiamen University, Xiamen 361005, China; 2 The Institute of Precision Machinery and Smart Structure, College of Engineering, Zhejiang Normal University, Jinhua 321004, China

## 1. Introduction

Microfluidics, also called lab-on-a-chip, is a cutting-edge technology in contemporary interdisciplinary science. Its development stems from the rapid progress of microelectronics manufacturing technology, as well as from the integration of multiple disciplines such as biomedicine, materials science, fluid mechanics, and interface science [1,2,3,4,5,6]. Its core technology involves constructing a chip system with micron-scale channels and structures to manipulate nanoliter or even picoliter fluids to complete a series of biochemical laboratory functions, including sample transmission, mixing, reaction, sorting, and detection [7,8,9,10,11,12,13,14]. At present, microfluidics has progressed from theoretical exploration to the forefront of clinical transformation and industrial application, playing an increasingly vital role in modern bioengineering.

The unique advantages of microfluidics technology show great potential in many research fields of bioengineering [15,16,17,18,19,20,21,22,23]. First, its highly integrated micro-systems effectively reduce sample usage and reagent consumption, significantly lowering experimental costs while enabling automated detection [24,25]. Secondly, its excellent control precision and rapid response speed facilitate real-time dynamic detection, making it particularly valuable for high-throughput single-cell analysis, rapid nucleic acid detection, and point-of-care testing (POCT) systems [26,27,28,29]. In addition, microfluidic technology can be flexibly designed into multi-channel and multi-modal information outputs, supporting comprehensive multi-parameter monitoring that provides crucial capabilities for precision medicine [30,31,32,33], early disease screening [34,35,36,37,38,39], and environmental toxicology detection [23,40,41,42,43].

In recent years, with the emergence of new materials and improved multi-physical-field coupling mechanisms, microfluidic technology has advanced toward intelligence, systematization, biomimicry, and clinical applications [44,45,46,47,48,49,50,51,52,53,54]. In microstructure manufacturing, traditional polydimethylsiloxane (PDMS) materials are being replaced by polymer materials characterized by biocompatibility, photoresponsiveness, and electrical responsiveness, creating more stable and reliable chip systems. For driving mechanisms, the integration of acoustic, electric, magnetic, and light fields enhances the flexibility of fluid manipulation while enabling precise control of biological cells and micro-nanoparticles [55,56,57,58]. The use of artificial intelligence algorithms for flow field modeling and image recognition has significantly improved system automation and data processing efficiency [59,60,61,62,63,64].

Against this background, this Special Issue of *Micromachines*, “Application of Microfluidic Technology in Bioengineering”, presents ten selected papers (seven interesting original articles and three reviews) from various research institutions, covering the latest progress of microfluidics in single-cell manipulation and detection, liquid biopsy, molecular diagnosis, interface design, and simulation modeling. These papers not only reflect the current multifaceted research landscape of microfluidics integration but also present the development trends in microfluidics at both the basic innovation and clinical application levels.

## 2. Overview of Published Articles

The eleven papers featured in this Special Issue cover five aspects of microfluidic technology, including single-cell manipulation and detection, liquid biopsy, molecular diagnosis, interface design, and simulation modeling.

### 2.1. Clinical Cell Analysis: Liquid Biopsy, Prenatal Testing, and Hematology 

In the field of cell manipulation and liquid biopsy, Chu et al. proposed a microfluidic chip system based on optically induced dielectrophoresis (ODEP) which achieved efficient, label-free enrichment and size-selective separation of circulating tumor cells (CTCs) [65]. This system relies on micromirror arrays to regulate spatial electric field distribution, enabling automatic screening of different-sized tumor cell subgroups in a very short time, with a separation purity reaching 93.5%. It has significant potential applications in the fields of liquid biopsy and early cancer screening. Yang et al. developed a self-assembled cell array chip (SACA) that combines multi-marker immunorecognition with microfluidic array design, achieving automated identification and extraction of fetal nucleated red blood cells (fnRBCs) [66]. The chip utilizes a joint discrimination strategy with markers such as Hoechst, CD71, and HbF to rapidly complete the extraction and paternity analysis of the extremely small number of fnRBCs in maternal blood, with a total operation time of less than 2 h and a sorting efficiency of 97.85%, providing a feasible new technical approach for non-invasive prenatal diagnosis. Hassan et al. conducted systematic modeling and experimental analysis on the impact of carrier fluid elasticity on cellular mechanical characterization in deformability cytometry [67]. They discovered significant errors in cell stiffness calculations when fluid viscoelastic effects are ignored. Simulation results based on CFD models indicate that fluid environment parameters significantly influence deformation test outcomes, suggesting that material rheological properties should be incorporated into future microfluidic chip structure optimization processes. Jiang et al. developed a microfluidic platform to evaluate red blood cell (RBC) deformability in healthy and thalassemia samples [68]. The chip, featuring capillary-mimicking microchannels, combined high-speed imaging with deep learning algorithms for automated cell tracking and contour analysis. The results confirmed significantly reduced deformability and slower recovery in thalassemic RBCs, especially in narrow channels, establishing a standardized and high-throughput method with strong diagnostic potential for hematological disorders.

### 2.2. Molecular Diagnostics and Extracellular-Vesicle Analysis

For molecular detection and nucleic acid analysis, Xian et al. proposed a MEMS digital PCR system based on superhydrophilic microarray structures, which can complete rapid, multi-target nucleic acid detection in reaction chambers as small as 120 pL [69]. The system features high integration and easy operation and enables multi-channel amplification without droplet encapsulation, demonstrating excellent detection sensitivity and specificity for clinically relevant markers such as hepatitis B virus and EGFR mutations, with significant practical value for point-of-care testing (POCT). Chen et al. authored a review systematically examining recent major strategies and technological platforms for exosome separation and analysis in microfluidics [70]. The article evaluates various approaches, including micro/nanopore arrays, acoustic chips, dielectric enrichment, and microbead capture, while pointing out the current contradiction between sample throughput and enrichment purity. It suggests that future systems could integrate electroacoustic synergistic strategies with AI-assisted data analysis to enhance overall system performance.

### 2.3. Interface and Materials Engineering for Robust Microfluidic

Regarding microfluidic chip interface design, Wu et al. employed a single-cycle brush-grafted polymer modification strategy to construct liquid bionic interfaces on PDMS surfaces, effectively enhancing the anti-fouling performance of microfluidic chips in complex blood environments [71]. Through droplet contact angle and fluorescent labeling experiments, this interface significantly inhibited protein adsorption and cell adhesion, improving system reusability stability and making it suitable for multiple sampling and analysis scenarios. From a fluid dynamics modeling perspective, Ayeni et al. analyzed the effects of microfiber bundles with different arrangements and densities on oil–water interface morphology and flow behavior [72]. The research revealed that controlling fiber spacing within the range of 10–50 μm can maximize interface area, helping to improve interface reaction efficiency and providing engineering optimization ideas for microfluidic extraction, emulsification, and phase separation process design.

### 2.4. System Modeling and Thermal Engineering

In the field of microfluidic chip system construction, Thiem et al. developed a thermal conduction model for a microfluidic cooling system based on a micro-hotplate substrate, addressing the needs of cryo-electron microscopy sample preparation [73]. The simulation results showed that when using a silicon-based substrate with a thickness of less than 5 μm, instantaneous cooling rates exceeding 10^6^ K/s can be achieved, potentially meeting the extreme freezing requirements for preserving biological macromolecular structures, thus providing theoretical support for organ-on-chip freezing processes and cell preservation technologies.

### 2.5. Methodological Reviews: Microfluidics-Enabled Omics and Impedance Sensing

The other two reviews in the Special Issue focus on the introductory biological applications of microfluidics. Sun et al. conducted a comprehensive review of the application of microfluidic technology in single-cell RNA sequencing and spatial omics research [74]. The article reviewed the microfluidic chip structure and application scenarios of technical platforms such as Drop-seq, 10× Genomics, and MERFISH, pointing out that microfluidics has shown extremely high value in revealing cell heterogeneity, reconstructing developmental lineages, and studying the tumor microenvironment. The authors look forward to the trend of spatial resolution improvement and multimodal collaborative development. Finally, Shen et al. focus on the basic principles, system construction, and biological applications of microfluidic impedance detection technology [75]. The review systematically introduces the cell equivalent circuit modeling, impedance spectrum feature extraction, and multi-parameter detection methods; sorts out the typical application cases of this technology in multiple dimensions, such as tumor cell identification, blood component analysis, and microbial monitoring; and proposes the future integration of flexible electronics and AI algorithms as a development direction.

## 3. Conclusions

The ten papers included in this Special Issue cover multiple areas, from modeling microscopic mechanisms to clinical transformations, reflecting the multi-dimensional capabilities of pathfinding and system construction in microfluidic technology for bioengineering applications. Whether in experimental design, material innovation, or analysis strategies and data interpretation, each research team has demonstrated solid technical skills and forward-looking application perspectives, highlighting the significant value of microfluidic technology in promoting medical intelligence, systematization, and individualization. Finally, we would like to highlight that this Special Issue collects cross-disciplinary and complementary contributions from China, the USA, and Germany, representing a shared and enhanced cross-technological research effort regarding microfluidic technology.

## Data Availability

Data underlying the results presented in this paper are not publicly available at this time but may be obtained from the authors upon reasonable request.
